# MicroRNAs Promote the Progression of Sepsis-Induced Cardiomyopathy and Neurovascular Dysfunction Through Upregulation of NF-kappaB Signaling Pathway-Associated HDAC7/ACTN4

**DOI:** 10.3389/fneur.2022.909828

**Published:** 2022-06-09

**Authors:** Qiancheng Luo, Hanning Ma, Enwei Guo, Lin Yu, Ling Jia, Bingyu Zhang, Gang Feng, Rui Liu

**Affiliations:** ^1^Department of Critical Care Medicine, Shanghai Pudong New Area Gongli Hospital, Shanghai, China; ^2^Department of Emergency Medicine, General Hospital of Ningxia Medical University, Shanghai, China

**Keywords:** neurovascular, biomarkers, HDAC7, ACTN4, microRNA, NF-kappaB (NF-κB), sepsis-induced cardiomyopathy (SIC)

## Abstract

**Introduction:**

The objective of this study was to determine the NF-kappaB pathway, hub genes, and transcription factors (TFs) in monocytes implicated in the progression of neurovascular-related sepsis-induced cardiomyopathy (SIC) as well as potential miRNAs with regulatory functions.

**Methods:**

: Sepsis-induced cardiomyopathy—and heart failure (HF)-related differentially expressed genes (DEGs) between SIC and HF groups were identified separately by differential analysis. In addition, DEGs and differentially expressed miRNAs (DEmiRNAs) in monocytes between sepsis and the HC group were identified. Then, common DEGs in SIC, HF, and monocyte groups were identified by intersection analysis. Based on the functional pathways enriched by these DEGs, genes related to the NF-kB-inducing kinase (NIK)/NF-kappaB signaling pathway were selected for further intersection analysis to obtain hub genes. These common DEGs, together with sepsis-related DEmiRNAs, were used to construct a molecular interplay network and to identify core TFs in the network.

**Results:**

: A total of 153 upregulated genes and 25 downregulated genes were obtained from SIC-, HF-, and monocyte-related DEGs. Functional pathway analysis revealed that the upregulated genes were enriched in NF-κB signaling pathway. A total of eight genes associated with NF-κB signaling pathway were then further identified from the 178 DEGs. In combination with sepsis-related DEmiRNAs, HDAC7/ACTN4 was identified as a key transcriptional regulatory pair in the progression of SIC and in monocyte regulation. hsa-miR-23a-3p, hsa-miR-3175, and hsa-miR-23b-3p can regulate the progression of SIC through the regulation of HDAC7/ACTN4. Finally, gene set enrichment analysis (GSEA) suggested that HDAC7/ACTN4 may be associated with apoptosis in addition to the inflammatory response.

**Conclusion:**

: hsa-miR-23a-3p, hsa-miR-3175, and hsa-miR-23b-3p are involved in SIC progression by regulating NF-κB signaling signaling pathway-related HDAC7/ACTN4 in monocytes and cardiac tissue cells. These mechanisms may contribute to sepsis-induced neurovascular damage.

## Introduction

Sepsis is a life-threatening organ dysfunction resulting from a dysregulated response of the organism to infection (1). Cardiac dysfunction caused by sepsis is defined as sepsis-induced cardiomyopathy (SIC) (2). The incidence and progression of SIC involve inflammatory responses, mitochondrial disorders, and metabolic changes (3). Impaired cardiac function occurs in approximately 60% of patients with septic shock within 3 days of admission (4). Patients with SIC have a poor prognosis and high morbidity and mortality rates (5). The mortality rate for patients with sepsis without cardiovascular compromise is 20% (6). However, the mortality rate increases for patients with SIC (7, 8). Severe toxic symptoms cause abnormal energy metabolism and myocardial damage in patients with sepsis, induce myocardial cell dysfunction, and eventually lead to severe events such as HF, which may endanger the patient's life (9–11). In recent years, SIC has also been found to cause dysfunction of the vascular nerve unit, which can lead to neurovascular dysfunction (12, 13). However, there is a lack of biomarkers and studies on the association of SCI with neurovascular diseases.

Researchers have found that the degree of cardiac dysfunction is a major factor in predicting mortality and morbidity in sepsis (14). The growing body of research confirms that signaling between the brain and circulatory system is essential to maintaining homeostasis during sepsis (15, 16). In patients with sepsis, endotoxin damages the cardiac and nervous systems (17). Endotoxins activate neutrophils excessively in sepsis, resulting in abnormal activation of the NF-kappaB signaling pathway; this interferes with the signaling communication between the brain and the immune system, contributing to vascular nerve damage associated with sepsis (12, 15). NF-kappaB-related signaling pathways are activated in the cardio-cerebral system; however, their molecular biological mechanisms need to be further elucidated (18). The NF-kappaB pathway can specifically block the proinflammatory and proapoptotic signaling caused by sepsis in the heart and brain, which can save other organs from the negative effects of sepsis (18, 19). Investigating the process of SIC and SIC-related neurovascular damage in the presence of the NF-kappaB-related signaling pathway is the focus of this study.

The activation of NF-kappaB signaling pathways is closely associated with monocytes in SIC (20). Monocytes are involved in various biological pathways that are crucial for the progression and prognosis of a disease. In addition, these pathways play a key role in the onset and progression of SIC. Monocytes are crucial regulators of inflammation. In response to inflammatory stimuli, monocytes are activated and migrate to the sites of inflammation to participate in the progression of inflammation (21). The inflammatory response causes upregulation of gene expression to trigger a massive release of inflammatory factors such as interleukin (IL)-6 and tumor necrosis factor (TNF)-α, leading to the incidence of SIC (2). Monocytes are also involved in oxidative stress (22). Intracellular over-activation of oxidative stress plays an important role in SIC (23). There are two immune responses in the course of sepsis, a hyperinflammatory response and immunosuppression (24). Death in patients with sepsis often occurs in the late immunosuppressive phase of the disease. The low expression of monocyte human leukocyte antigen-DR (mHLA-DR) is a universally recognized marker of an immunosuppressed state and is widely used in the treatment of sepsis (25–27). Therefore, identification of altered pathways in SIC and monocytes is essential to monitor the prognosis of patients with SIC.

microRNAs play a crucial regulatory role in the progression of various diseases (28–31). A study found that miR-21-3p is involved in the onset and progression of SIC (32). The miR-144-3p/NF-kB signaling pathway can regulate SIC injury (33). Furthermore, miR-133a-3p, miR-23b, and miR-155 are associated with SIC, suggesting that miRNA is a potential target for SIC therapy (34). In addition, miRNAs play a role in inflammation, oxidative stress, and apoptosis (35, 36), and these processes are also involved in SIC progression (37, 38).

Advancements in bioinformatics have enabled in-depth research into disease diagnosis and treatment from the perspective of big biological data (39–42). A large number of gene expression profiles can be easily obtained from RNA-sequencing (RNA-seq) data (43–46). High-throughput-based gene sequencing and functional pathway analysis allow bulk access to differentially expressed genes (DEGs) to examine the key pathways involved in disease progression (47, 48). This study aimed to identify the key pathways, hub genes, and TFs in monocytes and potential miRNAs with regulatory functions involved in the progression of SIC. In addition, we aimed to screen for targets and related neurovascular damage mechanism associated with the progression, diagnosis, treatment, and recurrence of SIC to monitor risks and eventually improve the prognosis of patients with SIC.

## Materials and Methods

### Data Acquisition and Pre-Processing

First, the keywords “Sepsis,” “Sepsis-induced cardiomyopathy (SIC),” “Septic cardiomyopathy (SCM),” and “Heart failure (HF)” were searched in the Gene Expression Omnibus (GEO) database. Datasets were filtered for bioinformatic analyses according to the following criteria: (1) data collected from human tissues; (2) single-cell RNA-sequencing (scRNA-seq) data; (3) sepsis and healthy control data; and (4) inclusion of at least 3 samples per group in the bulk RNA-seq transcriptome dataset. The GSE94717 (49), GSE101639, GSE79962 (50), and GSE167363 (51) datasets were eventually included. GSE94717 and GSE101639 contain miRNA transcript data obtained from the blood samples of 15 (12 patients with sepsis and 3 healthy controls) and 9 (6 patients with sepsis and 3 healthy controls) subjects, respectively (49). These datasets were used to screen for sepsis-related DEmiRNAs. GSE79962 is a dataset consisting of mRNA transcript data obtained from the heart tissue samples of 51 subjects (20 patients with HF, 11 healthy controls and 20 patients with SIC). Transcriptomic data from the GSE79962 dataset were used to identify SIC- and HF-related (differentially expressed genes) DEGs (50). Finally, to explore disease-related gene transcription patterns at the single-cell level, we obtained scRNA-seq data of 5 subjects (2 healthy controls and 3 patients with sepsis) from the GSE167363 dataset for differential analysis of gene expression at the single-cell level (51).

### Quality Control and Integration of ScRNA-Seq Data

As described in previous studies, RNA-seq data obtained from a total of 31,909 single cell from 5 samples were subjected to quality control using the “Seurat” package (52–55). The inclusion criteria for cells were as follows: (1) samples with 200–6,000 DEGs; (2) RNA counts > 1,000; (3) mitochondrial gene expression <20%; and (4) hemoglobin-related gene expression <1% (51). In addition, the inclusion criteria for cell characteristics were set as expression in at least 3 cells. A total of 30,091 cells and 20,597 characteristics were included in the subsequent single-cell analysis. Finally, the scRNA-seq data were integrated using the “SCTransform” function.

### Cell Clustering and Annotation

After integration of the scRNA-seq data, the “RunPCA” and “RunUMAP” functions were used to extract characteristics and reduce the dimensionality of single-cell transcripts. The “FindNeighbors” function was used to cluster the cells based on the default top 30 principal components (PCs), and the uniform manifold approximation and projection (UMAP) was used to visualize the cell clusters (56). Subsequently, the SingleR (version: 1.4) R package was used for cell cluster annotation based on the Monaco reference dataset (36). We calculated the number of cell types in each category as a percentage of the total number of cells in each sample.

### Differential Analysis of DEGs

At the level of scRNA-seq, monocytes were isolated to calculate monocyte-related DEGs in patients with sepsis (SP group) and healthy controls (HC group). The “FindMarker” function was used to identify monocyte-related DEGs, and the “ComBat” function was used to remove batch effects from different datasets before calculating DEmiRNAs. At the level of bulk-RNA analysis, the “limma” R package was used to identify SIC-related DEGs in the SIC and HC groups, HF-related DEGs in the HF and HC groups, and sepsis-related DEmiRNAs in the SP and HC groups at the individual level (57). *p*-Value < 0.05 was set as the threshold for DEG identification, and fold change values were used to identify upregulated and downregulated DEGs.

### Intersection Analysis

After identifying monocyte-, SIC-, and HF-related DEGs, intersection analysis was performed to identify common DEGs. Specifically, the analysis was performed separately for upregulated and downregulated DEGs, yielding co-upregulated or co-downregulated DEGs associated with monocytes, SICs, and HFs. Finally, the “VennDiagram” R package (https://cran.r-project.org/web/packages/VennDiagram/index.html) was used to draw a Venn diagram to present the results of the intersection analysis.

### Functional Pathway Analysis and Gene Set Enrichment Analysis

Common DEGs were subjected to the functional pathway analysis to identify pathways in which these dysregulated genes may be involved. The Kyoto Encyclopedia of Genes and Genomes (KEGG) and Gene Ontology (GO) analyses were performed using the “clusterProfiler” R package (58). GO terms are classified as biological process (BP), cellular component (CC), and molecular function (MF). We performed functional pathway analyses for co-upregulated and co-downregulated DEGs separately. Based on the “c2.cp.v7.2.symbols.gmt [Curated]” reference gene set, gene set enrichment analysis (GSEA) was performed to identify functional pathways enriched by the hub genes as reported in previous studies (55, 59).

### Downloading of Genes Associated With NF-κB Signaling Pathway

The results of the functional pathway analysis suggested that the NF-kB-inducing kinase pathway (NIK)/NF-kappaB signaling pathway (GO: 0038061) was upregulated in the monocytes of patients with sepsis, SIC, and HF. Therefore, we further retrieved and collected a list of 144 genes associated with this pathway from the Molecular Signatures Database (MSigDB) (59–62). Genes in this gene set were subjected to intersection analysis with common DEGs to obtain DEGs associated with NF-κB signaling pathway.

### Construction of a Molecular Interaction Network

From TRRUST (v2), a list of TF and target genes with corresponding expression levels was downloaded and used to predict TF-target gene pairs in DEGs. Then, miRWalk (< http://mirwalk.umm.uni-heidelberg.de/>), based on the identified sepsis-related DEmiRNAs, was used to identify predicted TF-target gene pairs (63). Among the predicted miRNA–mRNA interaction pairs, only those molecular pairs that were dysregulated in the opposite direction were retained. We then visualized the molecular interaction network using Cytoscape 3.5.1 based on these predicted TF-target genes and miRNA–mRNA pairs (64).

### Receiver Operating Characteristic Curve Analysis

The receiver operating characteristic (ROC) curve tool was used to assess the diagnostic capability of hub genes. In the horizontal and vertical coordinates of the curve, sensitivity was set on the y-axis, whereas “1-specificity” (i.e., false-positive rate) was set on the x-axis. Thereafter, the area under the ROC curve (AUC) was calculated to quantify the diagnostic capability. As an indicator of diagnostic capability, the AUC value should be usually between 1.0 and 0.5, with a value closer to 1.0, indicating a more accurate diagnosis. In addition, the ROC curve was plotted using the “pROC” package (65).

### Statistical Analysis

In this study, statistical analyses were performed and graphical plots were created using R (version 4.0.2). All tests were two-sided, and a *p*-value < 0.05 was considered significant.

## Results

### Quality Control and Integration of Single-Cell Data

**Figure 1:**
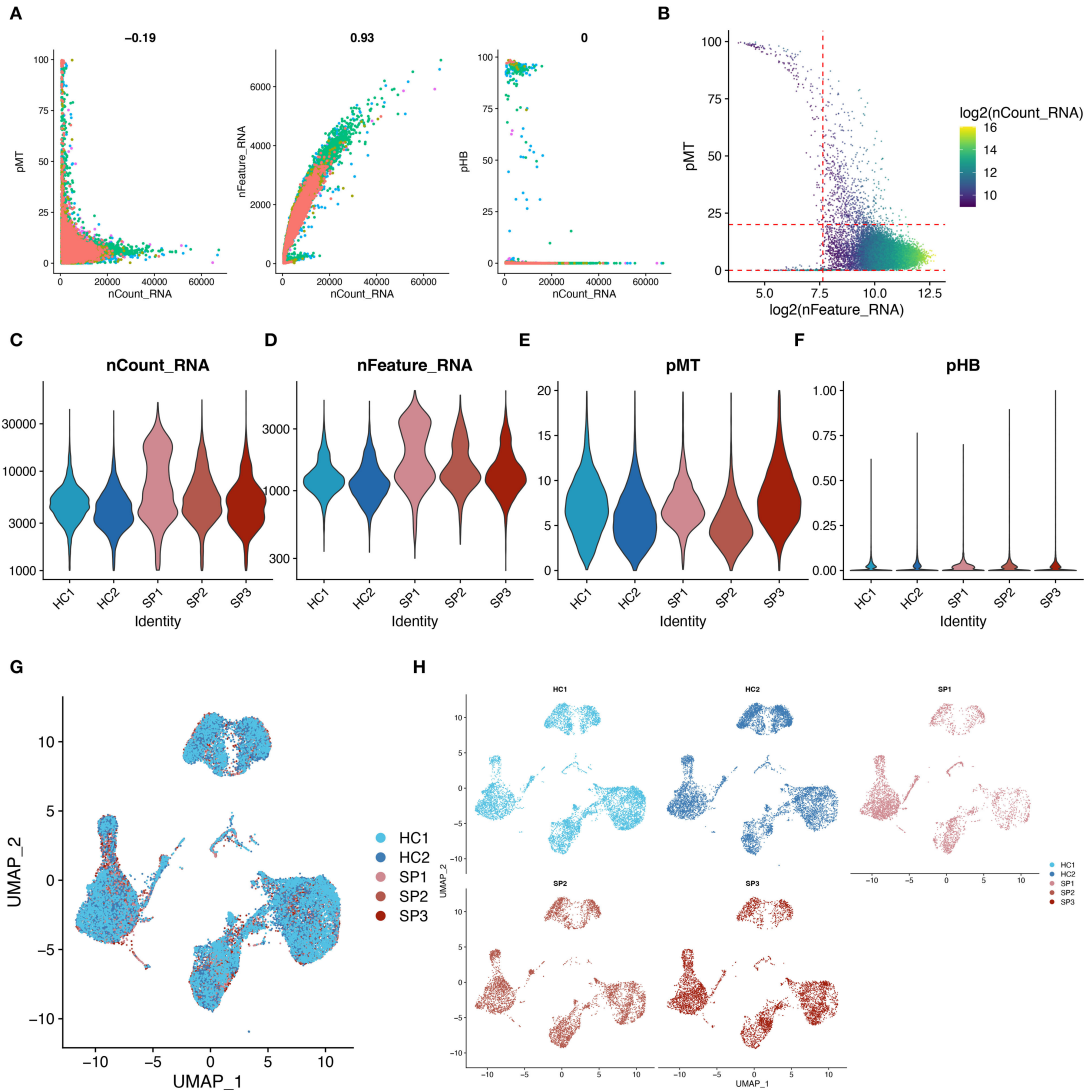
Quality control and data integration of single-cell datasets. **(A)** Scatter plot showing the relationship between cell characteristics in the single-cell data before data filtering. **(B)** Scatter plot showing the relationship between cell characteristics, mitochondrial proportions (pMT), and RNA counts. The red line represents the threshold used in performing cell mass filtering. **(C–F)** Violin plots showing the distribution of cellular features in each sample after data filtering. **(G,H)** UMAP showing the overlaps and distribution of single-cell data after integration. These plots show the removal of batch effects.

The distribution of key cell features, including feature counts, RNA counts, percentage of mitochondria (pMT), and percentage of hemoglobin (pHB), before quality control of the single-cell data, is shown in Figure 1A. Subsequently, the single-cell data were filtered with reference to the filtering conditions set in the original study from which the scRNA-seq data were obtained (Figure 1B) (51). The distribution of features of the filtered single-cell data is shown in Figures 1C–F. Subsequently, scRNA-seq data from five samples were integrated. Finally, UMAP showed that the batch effects among the five samples were removed, and the single-cell data were well integrated (Figures 1G,H). With the above quality control process, the quality of the data was assessed and low-quality cells were removed.

### Seven Cell Types Identified by Cell Clustering and Annotation

**Figure 2:**
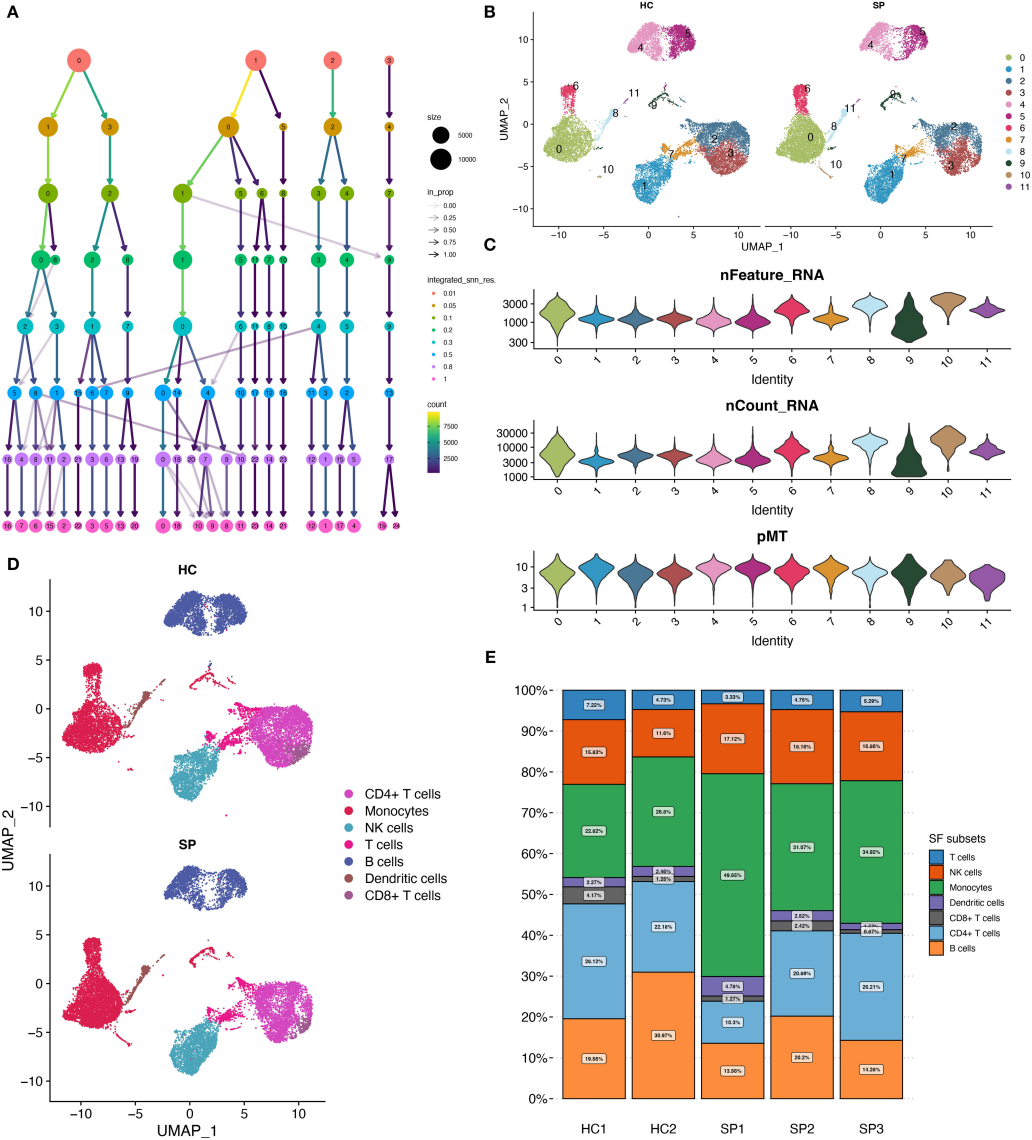
Cell clustering and annotation. **(A)** Diagram showing the cell clustering process. **(B)** UMAP showing the distribution of cell clusters, 0–11 indicate different subgroups of cells. **(C)** Cellular characteristics exhibited by each cell cluster. **(D)** UMAP showing the annotated cell types in HC and SP. **(E)** Relative proportion of each cell type between the five samples.

To examine the effects of optimal resolution on single-cell clustering, a range of resolution gradient values was selected (Figure 2A). Based on the relationship between the number of clusters and resolution, a final resolution of 0.3 was selected for clustering, which resulted in 11 cell clusters (Figure 2B). The distribution of RNA features, RNA count, and pMT in these 11 cell clusters is shown in Figure 2C. The features of different cell clusters were different, suggesting possible heterogeneity among the clusters. Subsequently, using the Monaco reference dataset, these cell clusters were identified as seven major cell populations, including monocytes, natural killer (NK) cells, T cells, B cells, dendritic cells, CD4+ T cells, and CD8+ cells (Figure 2D). The proportion of monocytes fluctuated from 22.82 to 49.65% in the five samples (Figure 2E). In the SP group, there were more monocytes than in the HC group (37.5 vs. 24.9%, respectively).

### Common DEGs Associated With Monocytes, SICs, and HFs

**Figure 3:**
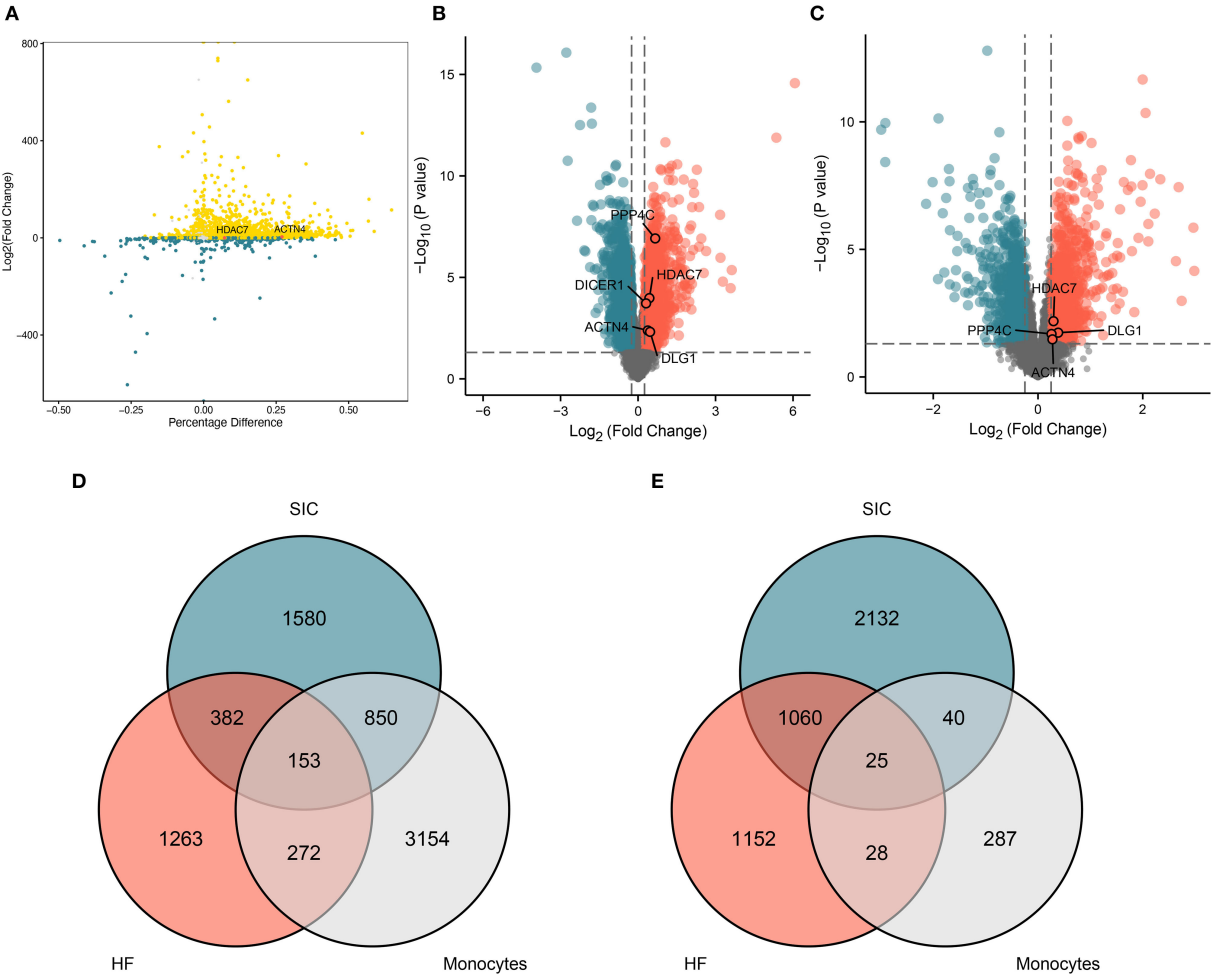
Genes co-differentially expressed. **(A)** The differentially expressed genes associated with monocytes in sepsis. **(B)** Volcano plot showing differentially expressed genes associated with SIC. **(C)** Volcano plot showing differentially expressed genes associated with HF. **(D,E)** Venn diagram showing the identified differentially expressed genes which are either **(D)** upregulated or **(E)** downregulated and are common in septic monocytes, heart failure, and septic cardiomyopathy; **(D,E)** shows 153 and 25 shared genes, respectively.

The differential expression analysis of DEGs associated with monocytes between the SP and HC groups yielded 4,429 upregulated and 380 downregulated DEGs (Figure 3A), which were named monocyte-related DEGs. SIC- and HF-related DEGs are shown in volcano plots (Figures 3B,C). The Venn diagram showed that a total of 153 genes were co-upregulated (Figure 3D) and 25 genes were co-downregulated in the monocyte, SIC, and HF groups (Figure 3E). These common DEGs were identified as hub genes involved in the progression of SIC. In addition, they were found to be dysregulated in monocytes during the onset and progression of sepsis. Therefore, these dysregulated genes may be the hub genes involved in the progression of SIC.

### DEGs Associated With NF-κB Signaling Pathway

**Figure 4:**
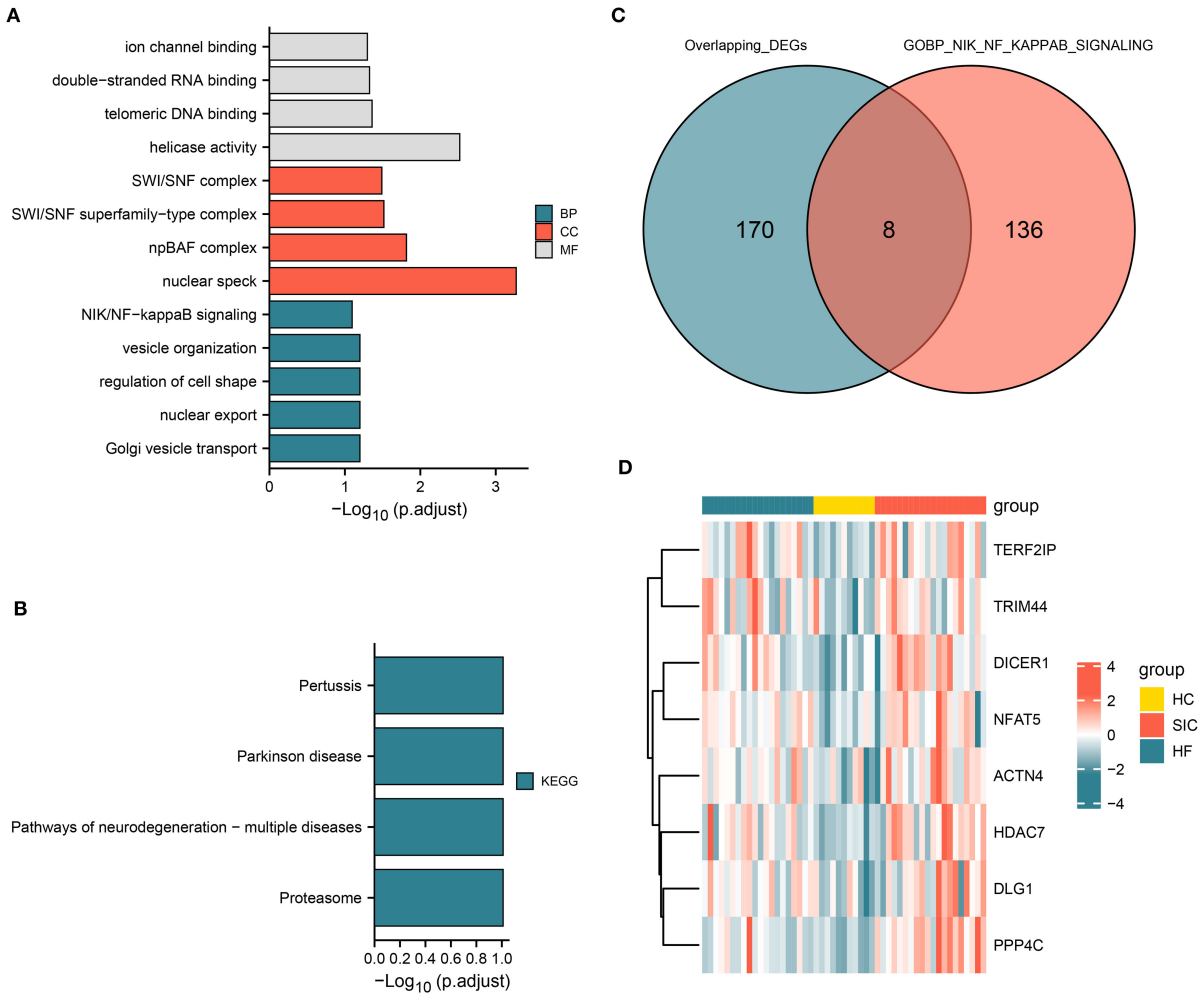
Functional pathway analysis and genes associated with the NIK/NF-kappa B signaling pathway. **(A)** Pathways associated with upregulated genes; molecular function (MF), cellular component (CC), and biological process (BP). **(B)** Pathways related to upregulated and downregulated genes. **(C)** Intersection of the differentially expressed genes and genes involved in the NIK/NF-kappa B signaling pathway. **(D)** Heatmap showing the intersecting genes in each group.

The functional pathway analysis showed that the identified co-upregulated genes were mainly enriched in NF-κB signaling pathway and pathways related to the regulation of cell shape (Figure 4A). The co-downregulated genes were mainly enriched in pathways associated with proteasome, Parkinson's disease, and pertussis (Figure 4B). Among these pathways, NF-κB signaling pathway is associated with various biological processes including immunity, inflammation, stress response, B-cell development, and lymphoid organogenesis. The intersection analysis of common DEGs and genes associated with NF-κB signaling pathway revealed eight dysregulated genes (e.g., *ACTN4, DICER1, DLG1, HDAC7, NFAT5, PPP4C, TERF2IP*, and *TRIM44*) (Figure 4C). The expression of these eight genes among the SIC, HF, and HC groups is shown in Figure 4D. These genes were upregulated in both SIC and HF groups, suggesting that they may be the hub genes involved in the progression of SIC *via* NF-κB signaling pathway.

### Role of HDAC7/ACTN4 Regulation in Monocytes in the Progression of SIC

**Figure 5:**
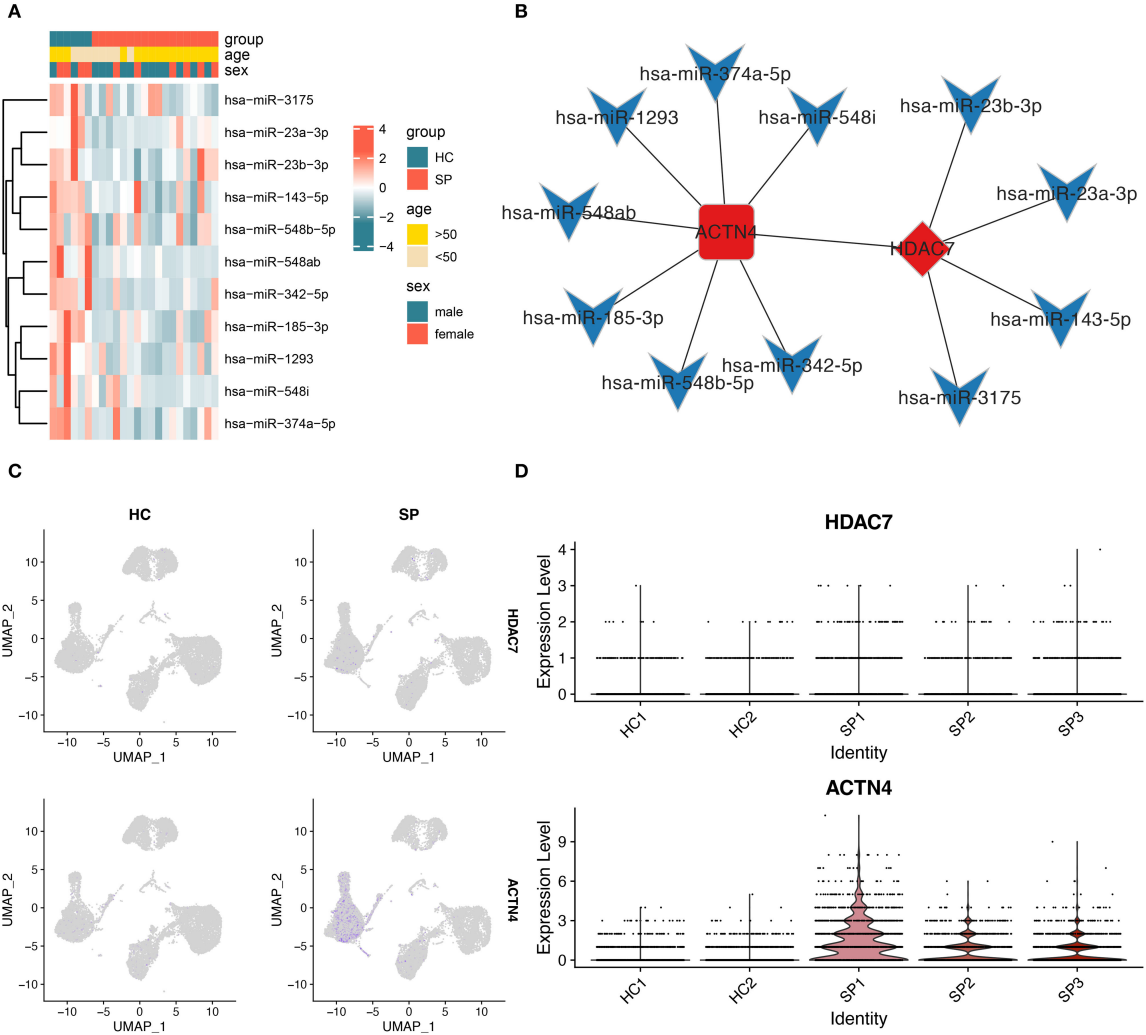
Construction of the molecular interaction network. **(A)** Heatmap showing the expression of miRNAs in the molecular interaction network between HC and SP groups. **(B)** Molecular interaction network showing HDAC7 as the core TF and *ACTN4* as the gene regulated by HDAC7. The diamond represents TFs, the round rectangle represents mRNAs, the V-shaped icon represents miRNAs, red bars indicate upregulated genes, and blue bars indicate downregulated genes. **(C)** Figure showing the expression of *HDAC7* and *ACTN4* between HC and SP groups. **(D)** Expression distribution of HDAC7 and ACTN4 between individual samples in single-cell data analysis.

To examine the molecular regulatory network that may play a crucial role in the progression of SIC, a molecular interaction network was constructed for sepsis-related DEmiRNAs and NF-κB signaling pathway-related DEGs. In this regulatory network, HDAC7 was found to be the transcriptional regulator of ACTN4. Figure 5A shows the expression profiles of 11 DEmiRNAs that may regulate ACTN4 and HDAC7. The results revealed 11 hub miRNAs, which, along with the HDAC7/ACTN4 regulatory pair, constitute the key molecular interaction network involved in SIC progression (Figure 5B). A total of four miRNAs had negative regulatory effects on HDAC7, whereas seven miRNAs had negative regulatory effects on ACTN4. The *p*-values and fold change distributions of the differential analysis of HDAC7 and ACTN4 were shown in the volcano plot (Figures 3B,C). In addition, UMAP showed that the expression of HDAC7 and ACTN4 in blood was mainly concentrated in monocytes (Figure 5C) and was higher in the SP group than in the HC group (Figures 3A, 5D). Therefore, we hypothesized that HDAC7/ACTN4 regulation in monocytes may play a crucial role in the progression and recurrence of SIC.

### Establishment of an MiRNA–TF–MRNA Regulatory Axis With ACTN4/HDAC7 as the Core

**Figure 6:**
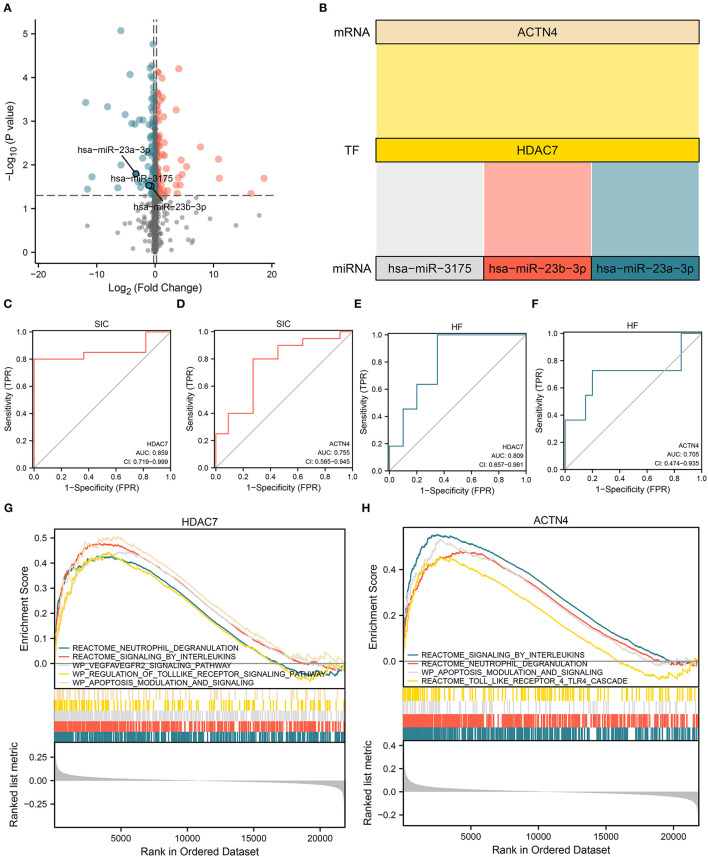
Establishment of the miRNA/TF/mRNA axis and gene set enrichment analysis (GSEA). **(A)** Volcano plot showing the differential expression of miRNAs that regulate TF in molecular interaction networks. **(B)** Sankey diagram showing the core miRNA/TF/mRNA regulatory axis. **(C,D)** ROC curves showing the ability of **(C)** HDAC7 (AUC:0.859) and **(D)** ACTN4 (AUC:0.755) to distinguish between SIC and HC. **(E,F)** ROC curves showing the ability of **(E)** HDAC7 (AUC:0.809) and **(F)** ACTN4 (AUC:0.705) to distinguish between HF and HC. **(G,H)** GSEA results for **(G)** HDAC7 and **(H)** ACTN4.

Based on the HDAC7/ACTN4 regulatory pair, three significant DEmiRNAs regulating HDAC7, including hsa-miR-23a-3p, hsa-miR-3175, and hsa-miR-23b-3p, were further identified (Figure 6B). The AUC values of HDAC7 and ACTN4 as the biomarkers for SIC diagnosis were 0.859 and 0.755, respectively (Figures 6C,D). For the diagnosis of HF/HC group, the AUC values for HDAC7 and ACTN4 were 0.809 and 0.705, respectively (Figures 6E,F). These results suggest that HDAC7 and ACTN4 have good predictive ability for the diagnosis of SIC and HF. Finally, three potential miRNAs regulating HDAC7 were identified with HDAC7/ACTN4 as the core, and the corresponding miRNAs–HDAC7-ACTN4 axis was established.

### Role of HDAC7/ACTN4 in Inflammatory Response and Apoptosis

Gene set enrichment analysis was performed to examine the potential role of dysregulated HDAC7 in the progression of SIC based on the bulk RNA transcript data of patients with sepsis. The results showed that the neutrophil degranulation, IL signaling, VEGFA–VEGFR2 signaling, and apoptosis modulation pathways were upregulated in samples with upregulated HDAC7 (Figure 6G). In samples with elevated ACTN4 expression levels, the interleukin signaling, neutrophil degranulation, and apoptosis modulation pathways were also upregulated (Figure 6H). Therefore, in addition to the inflammatory response, HDAC7/ACTN4 upregulation may also be involved in several pathways including apoptosis. Alterations in these pathways may be associated with the progression of SIC and HF, thus affecting patient prognosis.

## Discussion

In this study, bioinformatic analyses revealed the neurovascular-related NF-κB signaling pathway as the main pathway enriched by SIC-, HF-, and monocyte-related DEGs. NF-κB signaling pathway and genes in this pathway were screened from the enriched functional pathways. In addition, HDAC7 and ACTN4 were identified as the key genes involved in the progression of SIC *via* NF-κB signaling pathway. hsa-miR-23a-3p, hsa-miR-3175, and hsa-miR-23b-3p were identified as the possible regulators of SIC progression, which act by regulating HDAC7/ACTN4. Finally, HDAC7 and ACTN4 were subjected to GSEA, which suggested that HDAC7/ACTN4 were involved in apoptosis as well as inflammation.

A previous study found that the NF-kB signaling pathway regulates SIC injury (33). In the early stages of sepsis, bacterial stimulation can cause significant changes in the NF-kB-inducing kinase (NIK) pathway (66). The inflammatory factor IL-33 can increase pyroptosis levels in macrophages and mortality in septic mice by activating the NF-kB signaling pathway (67). Moreover, NF-kB affects the inflammatory process in various diseases such as asthma and kidney diseases (68–70). In addition, oxidative stress generated by NF-kB-induced iNOS and COX-2 signaling pathways can impair myocardial function in patients with sepsis (71, 72). NF-kB mediates the transcription of several proinflammatory genes and induces the release of inflammatory factors such as ILs and TNF-α, which leads to myocardial dysfunction and accelerates the progression of SIC (73). Drugs targeting NF-kB inhibition can help to improve cardiac function in patients with SIC and hence improve their prognosis (73). Therefore, NF-kB pathway-related genes may play a crucial role in the onset and progression of SIC. Dysregulated monocytes may produce large amounts of inflammatory cytokines, resulting in widespread inflammation, organ failure, and even death (51). Furthermore, monocytes are involved in the release of inflammatory mediators (24). Circulating monocytes in HF are also pathologically activated through enhanced NF-κB activity (74–76). Therefore, monocytes are closely associated with the progression of SIC and the onset of HF.

In this study, HDAC7 and ACTN4 were identified, for the first time, as the hub genes involved in the progression of SIC *via* NF-κB signaling pathway. In addition, HDAC7/ACTN4 was upregulated in monocytes in patients with sepsis. A previous study reported a significant elevation in HDAC7 mRNA expression in the monocytes of patients with coronary artery disease (77). In addition, mCRP-treated monocytes have upregulated ACTN4 (78). The extracellular ACTN4-derived fragment has monocyte chemotactic activity and can promote monocyte maturation (79). Monocytes are involved in various biological processes such as inflammatory responses, oxidative stress, and immunosuppression (21, 25, 58), and these processes also influence the progression of SIC (23, 24, 37, 38, 59). We hypothesized that NF-κB-induced upregulation of HDAC7/ACTN4 in monocytes may be associated with the progression of SIC.

Studies reporting on the role of HDAC7/ACTN4 in sepsis or SIC are limited. However, a previous study found that HDAC7 is involved in the regulation of apoptosis (80). The enzymatic activity of HDAC7 is essential for TLR-induced production of inflammatory mediators and is involved in the inflammatory response (81). ACTN4 is involved in the inflammatory or immune response in the lungs (82). ACTN4 phosphorylation also mediates cell injury (83). Inflammatory response and apoptosis are involved in the progression of SIC (23, 37, 38). Similar to previous studies, the hub genes identified in this study, HDAC7 and ACTN4, were found to be associated with the inflammatory response.

By regulating the translation of HDAC7, hsa-miR-23a-3p, hsa-miR-3175, and hsa-miR-23b-3p may influence the inflammatory response in SIC and the extent of apoptosis. miR-23a-3p is one of the abundant miRNAs in the myocardial tissue, which attenuates apoptosis in myocardial cells during ischaemia–reperfusion injury (84). It is closely related to the incidence of myocardial lesions and HF (85). Downregulation of miR-23a-3p expression in acute HF promotes polarization of macrophages toward the repair phenotype (86). In addition, miR-23a-3p reduces superoxide dismutase-induced oxidative stress injury (49). Oxidative stress is an important biological process in the progression of SIC (23). miR-23b inhibits SIC progression by attenuating the inflammatory response, suppressing apoptosis, and blocking NF-κB activation and is a potential target for SIC therapy (34). Previous studies on miR-3175 have focused on tumors (87, 88). However, miR-3175 is also involved in oxidative damage of cells (89). Based on the results of this study, we hypothesized that hsa-miR-23a-3p, hsa-miR-3175, and hsa-miR-23b-3p play a regulatory role in the progression of SIC by interfering with HDAC7/ACTN4 in monocytes and cardiac tissue cells.

The neutrophil degranulation, IL signaling, VEGFA–VEGFR2 signaling, and apoptosis modulation pathways were upregulated in samples with elevated HDAC7 expression levels. These results suggest that in addition to the inflammatory response, upregulation of HDAC7/ACTN4 may also be involved in some apoptosis-related pathways. A previous study also reported the involvement of apoptosis in the progression of SIC (90). Therefore, HDAC7/ACTN4 may be involved in pathways related to inflammatory response and apoptosis, thus influencing the recovery, recurrence, and progression of SIC and affecting the prognosis of patients with SIC. SIC-, HF-, and monocyte-related DEGs are enriched in NF-κB signaling pathway. Moreover, hsa-miR-23a-3p, hsa-miR-3175, and hsa-miR-23b-3p may influence SIC progression by regulating HDAC7/ACTN4. These hub genes, TFs, and miRNAs may be the potential targets related to the progression, treatment, and recurrence of SIC. In addition, they can be used to monitor the risk of SIC and to improve the prognosis of patients with SIC. However, the results of this study were not validated in clinical samples, and relevant cellular and animal experiments were lacking. Moreover, we did not examine the relationship between HDAC7/ACTN4 and corresponding miRNAs in SIC further. Further studies are required to investigate the regulatory role of HDAC7/ACTN4 in the progression of SIC.

## Conclusion

Sepsis-induced cardiomyopathy in blood mononuclear cells and cardiac tissue cells is stimulated by serum levels of biomarkers (hsa-miR-23a-3p, hsa-miR-317, and hsa-miR-23b-3), which alter neurovascular-related HDAC7/ACTN4 signaling pathways linked to the NF-κB pathway. This study investigated the underlying mechanisms of neurovascular dysfunction associated with SIC and sepsis.

## Data Availability Statement

The original contributions presented in the study are included in the article/supplementary material, further inquiries can be directed to the corresponding author.

## Ethics Statement

Ethical review and approval was not required for the study on human participants in accordance with the local legislation and institutional requirements. Written informed consent from the patients/participants or patients/participants legal guardian/next of kin was not required to participate in this study in accordance with the national legislation and the institutional requirements.

## Author Contributions

QL: software, validation, formal analysis, data curation, reviewing and editing, and writing—original draft. RL: conceptualization, methodology, supervision, funding acquisition, writing, reviewing, and editing. HM: methodology, conceptualization, project administration, funding acquisition, and software. EG: conceptualization, project administration and data curation. LY: software, data curation, and supervision. LJ and GF: data curation, writing, reviewing and editing, and data curation. BZ: methodology, supervision, funding acquisition, and data curation. All authors contributed to the article and approved the submitted version.

## Funding

The study was funded by Clinical Characteristic Discipline Construction Project of Shanghai Pudong New Area Health Commission (PWYts2021-17).

## Conflict of Interest

The authors declare that the research was conducted in the absence of any commercial or financial relationships that could be construed as a potential conflict of interest.

## Publisher's Note

All claims expressed in this article are solely those of the authors and do not necessarily represent those of their affiliated organizations, or those of the publisher, the editors and the reviewers. Any product that may be evaluated in this article, or claim that may be made by its manufacturer, is not guaranteed or endorsed by the publisher.

## Abbreviations

SIC: Sepsis-induced cardiomyopathy
GEO: Gene Expression Omnibus
DEGs: Differentially expressed genes
DEmiRNAs: Differentially expressed microRNAs
DEmRNAs: Differentially expressed mRNAs
GO: Gene Ontology
KEGG: Kyoto Encyclopedia of Genes and Genomes
GSEA: Gene set enrichment analysis
scRNA-seq: Single-cell RNA sequencing.
